# Does Traumatic Brain Injury Lead to Criminality? A Whole-Population Retrospective Cohort Study Using Linked Data

**DOI:** 10.1371/journal.pone.0132558

**Published:** 2015-07-14

**Authors:** Peter W. Schofield, Eva Malacova, David B. Preen, Catherine D’Este, Robyn Tate, Joanne Reekie, Handan Wand, Tony Butler

**Affiliations:** 1 Neuropsychiatry Service, Hunter New England Local Health District, Newcastle, NSW, Australia; 2 Centre for Translational Neuroscience and Mental Health (CTNMH), University of Newcastle, Newcastle, NSW, Australia; 3 Centre for Health Services Research, School of Population Health, University of Western Australia, Perth, WA, Australia; 4 National Centre for Epidemiology and Public Health, Australian National University, Canberra, ACT, Australia; 5 Rehabilitation Studies Unit, University of Sydney, Sydney, NSW, Australia; 6 Kirby Institute, UNSW Australia, Sydney, NSW, Australia; University of Wuerzburg, GERMANY

## Abstract

**Background:**

Traumatic brain injury (TBI) may be a risk factor for criminal behaviour however multiple factors potentially confound the association.

**Methods:**

Record linkage and Cox proportional hazards regression analyses were used to examine the association between hospital-recorded TBI (n = 7,694) and subsequent first criminal conviction in a retrospective cohort matched 1:3 with 22,905 unaffected community controls and full-sibling controls (n = 2,397). Aboriginality, substance abuse, social disadvantage, and mental illness were included in analyses as potential confounders

**Results:**

In multivariable models, relative to general population controls, TBI was associated with any conviction (males: Hazard Ratio (HR) = 1·58 (95% CI 1·46 to 1·72); females: HR = 1·52 (95% CI 1·28 to 1·81)); and similar Hazard Ratios were obtained for the sibling analyses in males (HR = 1.68 (95% CI 1.31-2.18)) and females (HR 1.27 (95% CI 0.71-2.29)). TBI was also associated with violent convictions relative to the general population, (males: HR = 1.65 (95% CI 1.42 to 1.92); females HR = 1.73 (95% CI 1.21 to 2.47)), and in analyses with sibling controls in men (HR = 1.89 (95% CI 1.20-3.00)), but not in women (HR 0.73, 95% CI 0.29-1.81)).

**Conclusion:**

The results support a modest causal link between TBI and criminality after comprehensive adjustment for confounding. Reducing the rate of TBI, a major public health imperative, might have benefits in terms of crime reduction.

## Introduction

Studies of offender populations consistently show high rates of past traumatic brain injury (TBI) [1] and some investigators suggest that many offences may be a consequence of TBI-related behavioural dysregulation [2]. The societal, public health, criminological, and custodial implications of an established causal association between TBI and subsequent offending would be profound if, as the investigators of one study proposed, ‘a head injury leads victims to participate in more than half of the crimes that come to the attention of police and that result in incarceration’ [2]. With increasing concerns expressed about TBI among soldiers returning from conflicts such as Afghanistan and in those engaged in body contact sports a rigorous examination of the purported link between TBI and criminality is relevant, timely and important [3–4].

Most previous studies bearing on this issue have been cross-sectional [1–2, 5] but two large longitudinal studies using record linkage have also been published. In one, TBI in childhood was associated with a 1·7-fold increased risk of criminal conviction later in life relative to general population controls [6]. In the other, TBI leading to hospitalisation was associated with a 3-fold increased risk of a conviction for a violent offence relative to general population controls and a two-fold increased risk relative to non-exposed siblings [7]. More recently, results from a longitudinal birth cohort of children born in Christchurch New Zealand, indicated an association between TBI and self-reported arrests, property offenses and violent offenses [8]. For the current longitudinal retrospective cohort study, we use linked data and account for important confounders not comprehensively examined in these previous studies to obtain unbiased estimates of the association between a hospital-documented TBI and first criminal conviction, including a conviction for violence specifically [9–10].

## Methods

### Study sample

We used whole-population data linkage to identify individuals born in Western Australia (WA) between 1980–1985 inclusive who had attended a hospital in WA with record-evidence of a TBI. For each such individual, we randomly selected three individuals of the same sex and year of birth born in WA who had never been admitted to hospital for TBI (‘population controls’). We also identified all same-sex full-siblings without record-evidence of TBI born within +/- 5 years of the TBI-affected proband to serve as controls. When more than one suitable sibling was identified, the one nearest in age to their exposed counterpart was selected for the analyses.

### Evidence of TBI

Evidence of TBI was defined as a hospital admission with a primary admitting or additional diagnosis for any of the TBI diagnostic codes (S1 Table) [11–12].

### Other risk factors

Proxies for potential confounders including social disadvantage, mental illness, Aboriginality, and substance abuse were obtained as follows. A measure of socioeconomic disadvantage was derived from the Index of Relative Social Disadvantage from the Australian Bureau of Statistics (ABS) [13], ascertained at a postcode level for usual residence at the time of birth. Aboriginal descent was defined by the presence of at least one record across all available datasets identifying the person as Aboriginal [14]. Ever had a mental health problem was defined as having a mental health-related hospital admission or public outpatient mental health service contact according to ICD diagnostic codes: ICD-10 ‘F00’-‘F99’ or ICD-9 ‘290’-‘319’. Presence of a drug record was defined as having received a drug- or alcohol-related treatment episode recorded by the WA Drug and Alcohol Office.

### Definition of outcome

The outcome of interest was an incident criminal conviction as determined by WA Department of Corrective Service’s records, including juvenile offending (up to 2009) and adult offending up to September 2011 resulting in a criminal conviction by the WA Court system. This included community-based orders and custodial sentences. Additional analyses examined convictions for violent offences only, comprising homicide, assault (including sexual assault), robbery, or murder. In Australia, criminal responsibility begins from the age of 10 and we therefore excluded anyone who died before reaching this age. Participants with a criminal conviction prior to their first-time TBI were excluded from the study.

### Datasets

We used de-identified whole-population administrative data linked across the WA Departments of Health and Corrective Services, and the WA Drug and Alcohol Office. Data from the ABS were used to assign geocodes to postcodes provided in midwives’ notifications data. The State Health datasets included all midwives’ notifications, hospital separation records, death registrations, public outpatient and inpatient mental health records, and drug and alcohol treatment records. For our study, the midwives’ notification data contained information for all registered births in WA from 1980–1985, and for siblings until 1990. This information was used to identify each individual’s year and month of birth and sex. The hospital morbidity data contained information on all public and private hospital separations (discharges or deaths) in WA with corresponding diagnostic and procedural information coded according the ICD coding system from 1980–2010 [11–12]. Data from the WA Mortality Register were extracted for all deaths in WA from 1980–2011 for the study sample. Mental health data included information on mental health inpatient and publicly-funded outpatient admissions from 1980–2010. The WA Drug and Alcohol Office included information for all drug and alcohol treatment episodes recorded by the Office from 1980–2006. The WA Corrective Services datasets included juvenile (1993–2009) and adult (1997–2011) records as well as data on community corrections orders (i.e. non-custodial sentences). The Australian Census data contain information on neighbourhood economic disadvantage. All datasets were linked by the WA Data Linkage Branch at the WA Department of Health using established best-practice probabilistic matching protocols [15–16]. No other source of medical or judicial information was available to us; the datasets used were comprehensive with minimal missing data.

### Statistical Analysis

We included only individuals with their first conviction after the first diagnosis of TBI, and those who survived a full year from their first TBI to allow adequate time for an outcome to occur. To ensure consistency of risk periods for TBI-exposed and unexposed groups, controls were assigned a pseudo-TBI date based on the age at which the TBI occurred in the matched exposed cohort member and only controls without a conviction prior to that assigned date and who survived until the age of 10 and for 12 months following their assigned TBI date were included. All individuals were followed from their TBI date (pseudo or actual) until their first conviction, death, or 31^st^ December 2006, whichever occurred first. Males and females were analysed separately. Additional analysis looking at violent convictions only followed individuals until their first conviction for a violent offence, death, or 31^st^ December 2006.

Aboriginal descent, disadvantaged background, mental illness, treatment for substance abuse disorders and conviction records were compared between TBI and controls using the Chi-square test.

Univariable and multivariable Cox proportional hazard regression models were used to assess the association between TBI and risk of criminal conviction. Covariates including treatment for drug or alcohol abuse, mental illness, Aboriginality, socioeconomic disadvantage and year of birth were included in adjusted analysis.

The proportionality assumptions of the hazard functions were assessed for individual covariates and globally using Schoenfeld´s residuals [17]. At the global level, the proportionality assumption was not violated in any of the models considered (p-values in multivariate models were ranged from 0.1115–0.1725); however at the individual level, the proportionality assumption was violated for indigenous status in females (p-value = 0.0364). Following published guidelines [18], we stratified our analysis on the non-proportional predictor, indigenous status, and obtained estimates within each stratum indicating overall goodness of the fit of the final model. The final model fitted the data reasonable well except for the adjusted logistic regression in twins (males and females); and results for these analyses were excluded as the models did not converge. Proportionality assumptions on the hazards functions were also assessed for all the other subgroup analyses; data supported the proportionality assumptions on the hazard ratios: goodness of fit of the final models were at acceptable levels. Further evaluation using the Cox-Snell residuals provided additional reassurance that the models adopted fitted the data satisfactorily [18].

For analyses of siblings/twins analysis, univariable and multivariable stratified Cox regression models were used with separate baseline hazard functions across the families [17]. We undertook separate analyses for siblings and twins. Data were analysed using SAS Version 9·3 (SAS Institute Inc., Cary, NC, USA) and STATA 10·0 (College Station, TX, USA) using a two-tailed 5% significance level. Since Aboriginal descent, disadvantaged background, mental illness and substance abuse disorders have been associated both with conviction rates and TBI [1–2, 5–10], and are also potential effects modifiers, we included proxies for these factors as covariates and assessed their interactions with TBI, and also adjusted for year of birth.

### Ethics Statement

The protocol was approved by the Department of Health WA, Western Australian Aboriginal Health Ethics Committee (WAAHEC), Curtin University, and the WA Department of Corrective Services Research and Evaluation Committee. The data collected was de-identified and was therefore analysed anonymously.

## Results

**Fig 1:**
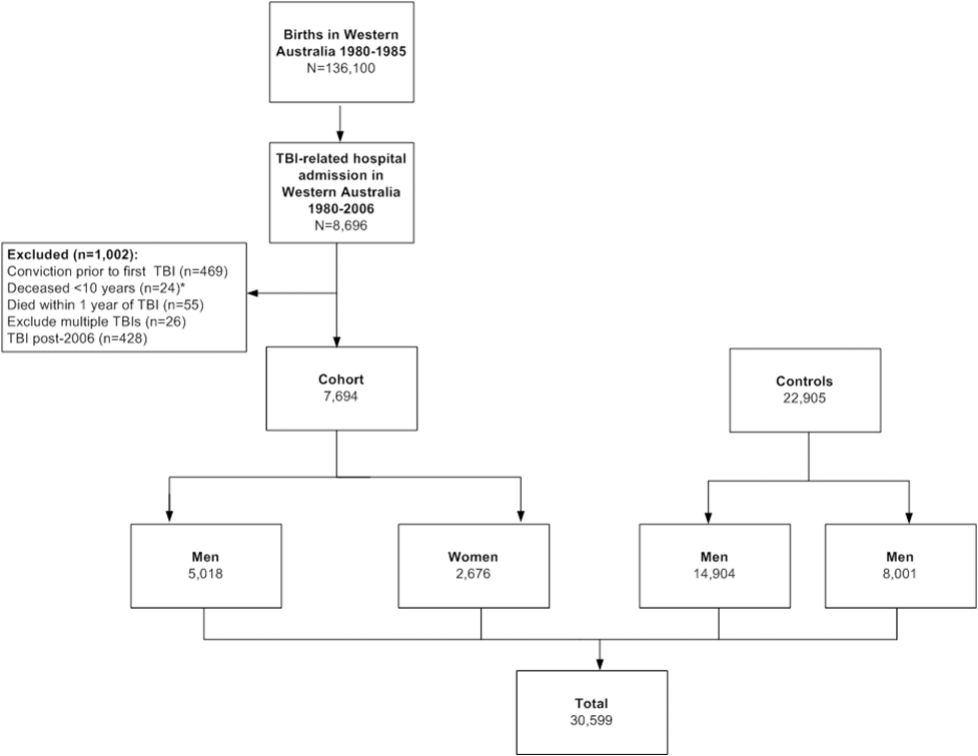
Overview of Sampling. Flowchart depicts the participant numbers that were included and excluded. *10 years is considered the age of criminal responsibility in Australia.

Of the total 136 100 individuals born from 1980–1985, 8242 were initially identified as having sustained a TBI resulting in a hospital admission in WA from 1980–2006. We excluded 469 convicted before the date of their TBI, 24 who died before the age of 10 years, and 55 who died within one year of their incident TBI (94% of whom died within two months of their TBI) leaving 7694 individuals in the cohort (5018 males, 2676 females,) (Fig 1). The median age at first TBI was 10·6 years in males (inter quartile range [IQR] 4·6–16·2) and 6·9 years in females (IQR 2·8–14·1). The medial follow-up time was 12·5 years for males (IQR 6·8–18·6) and 16·6 years for females (IQR 9·4–20·8).

Characteristics of the study groups are shown in S2 and S3 Tables. Relative to non-exposed community controls, those with a TBI had an approximately two fold rate of a conviction record (males: 18% vs 10%, p<0·0001; females: 9% vs 4%, p <0·0001), mental illness (males:19% vs 9%, p<0·001; females:22% vs 13%, p<0·001), and Aboriginal descent (males: 11% vs 6%, p<0·0001; females: 17% vs 6%, p<0·001).

Compared with community controls in unadjusted analyses TBI increased the risk of any conviction in males (Hazard Ratio (HR) = 1·85 (95% confidence interval (CI) 1·70 to 2·01)), and females (HR = 2·20 (95% CI 1·86 to 2·59)) (Table 1).

**Table 1:** Adjusted Hazard Ratios (95% CI) of Males (n = 19,992) and Females (n = 10,677) having a “Corrective Services Record” (CSR).

Groups	Sub-Groups	Overall [n(%)]		CSR [n(%)]		Adjusted Hazard Ratio (95% CI)		*p*-value	
		Male	Female	Male	Female	Male	Female	Male	Female
**Traumatic Brain Injury**	Not exposed	14904 (75)	8001 (75)	1554 (10)	332 (4)	1	1		
	Exposed	5018 (25)	2676 (25)	920 (18)	238 (9)	1·58 (1·46 to 1·72)	1·52 (1·28 to 1·81)	<0·001	<0·001
**Drug and alcohol treatment record** ^1^	No	19844 (99)	10628 (99)	2457 (12)	556 (5)	1	1		
	Yes	79 (0·4)	49 (0·46)	17 (22)	14 (29)	1·96 (1·21 to 3·17)	5·62 (3·27 to 9·67)	0·006	<0·001
**Mental illness diagnosis** ^1^	No	17611 (88)	9056 (85)	2146 (12)	448 (5)	1	1		
	Yes	2311 (12)	1621 (15)	328 (14)	122 (8)	1·10 (0·97 to 1·23)	1·23 (1·01 to 1·52)	0·145	<0·001
**Indigenous status**	No	18508 (93)	9705 (91)	1780 (10)	330 (3)	1	1		
	Yes	1414 (7)	972 (9)	694 (49)	240 (25)	4·98 (4·53 to 5·48)	6·52 (5·41 to 7·85)	<0·001	<0·001
**Index of Disadvantage** ^2^	Lowest	4801 (24)	2588 (24)	421 (9)	80 (3)	1	1		
	Low	4832 (24)	2574 (24)	489 (10)	124 (5)	1·01 (0·89 to 1·15)	1·31 (0·98 to 1·73)	0·848	0·065
	High	4921 (25)	2626 (25)	610 (12)	118 (4)	1·16 (1·02 to 1·31)	1·08 (0·81 to 1·43)	0·024	0·620
	Highest	4678 (24)	2509 (24)	885 (19)	233 (9)	1·42 (1·26 to 1·61)	1·54 (1·17 to 2·01)	<0·001	0·002
	Missing	690 (4)	380 (4)	69 (10)	15 (4)	0·85 (0·65 to 1·10)	1·03 (0·59 to 1·78)	0·197	0·926
**Year of Birth**	-	-	-	-	-	1·03 (1·01 to 1·05)	1·01 (0·96 to 1·06)	0·015	0·619

^1^ counted as “0” if occurred after the conviction

^2^ Highest index of disadvantage represents the lowest level of socioeconomic status (SES)

**Fig 2:**
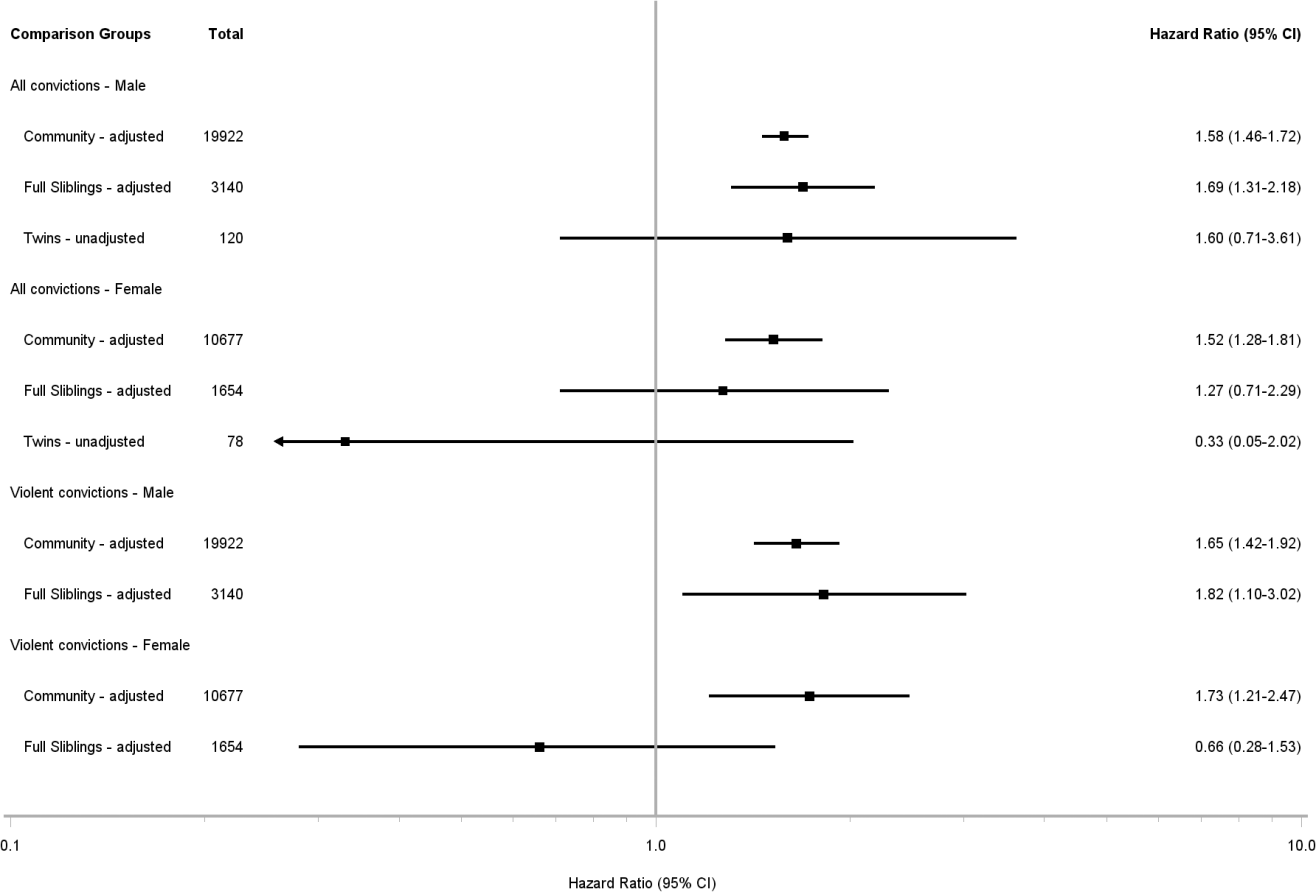
Forest Plot of Unadjusted and Adjusted Hazard Ratios for Any and Violent Convictions. Figure depicting the associations between TBI and any violent offending, relative to the non- exposed comparison groups: general community, full siblings, or twins.

In multivariable analyses there were no significant interactions between TBI and other risk factors for conviction and, compared with community controls, TBI was associated with increased risk for all offending in males (HR = 1·58 (95% CI 1·46 to 1·72)) and females (HR = 1·73 (95% CI 1·21 to 2·47)).When same-sex full-sibling controls were used in the adjusted analyses, increased risk of offending was evident among TBI-exposed males (HR = 1·69 (95% CI 1·31–2.18)) but was not significant in females (HR = 1·27 (95% CI 0·71 to 2.29)). In twins discordant for TBI exposure, models adjusted for covariates did not converge but in unadjusted analyses, males (HR = 1.60, (95% CI0.71–3.61)) but not females (HR = 0.33 (95% CI 0.05–2.02)) had a positive HR, in neither case statistically significant (Fig 2). For both sexes, increased hazard of offending was associated with a mental health record, drug and alcohol record, Aboriginality, and increased social disadvantage.

For violent convictions, relative to the general community, TBI was also associated with increased risk in men (HR = 1·65 (95% CI 1·42 to 1·92)) and women (HR = 1.73 (95% CI 1.21 to 2.47)) (Table 2). Among full siblings discordant for TBI, TBI was associated with violent offending in men (HR = 1.82 (95% CI 1.10–3.020)) but not in women (HR = 0.66 (95% CI 0.28–1.53)). There were insufficient data to conduct meaningful analyses with respect to violent convictions amongst twins for either sex (S4 Table).

**Table 2:** Adjusted Hazard Ratios (95% CI) of Males (n = 19,992) and Females (n = 10,677) having a “Violent Offence Record” (VOR).

Groups	Sub-Groups	Overall [n(%)]		VOR [n(%)]		Adjusted Hazard Ratio (95% CI)		*p*-value	
		Males	Females	Males	Females	Males	Females	Males	Females
**Traumatic Brain Injury**	Not exposed	14904 (75)	8001 (75)	404 (3)	64 (0.80)	1	1		
	Exposed	5018 (25)	2676 (25)	303 (6)	66 (2)	1·65 (1·42 to 1·92)	1·73 (1·21 to 2·47)	<0·001	0·002
**Drug and alcohol treatment record** ^1^	No	19844 (99)	10628 (99)	702 (4)	126 (1)	1	1		
	Yes	78 (0·4)	49 (0·46)	5 (6)	4 (8)	1·79 (0·74 to 4·35)	6·55 (2·34 to 18·30)	0·197	<0·001
**Mental illness diagnosis** ^1^	No	17611 (88)	9056 (85)	591 (3)	96 (1)	1	1		
	Yes	2311 (12)	1621 (15)	116 (5)	34 (2)	1·37 (1·12 to 1·68)	1·44 (0·96 to 2·17)	0·002	0·076
**Indigenous status**	No	18508 (93)	9705 (91)	360 (2)	46 (0·47)	1	1		
	Yes	1414 (7)	972 (9)	347 (25)	84 (9)	10·25 (8·73 to 12·04)	15·55 (10·48 to 23·07)	<0·001	<0·001
**Index of Disadvantage** ^2^	Lowest	4801 (24)	2588 (24)	68 (1)	11 (0·43)	1	1		
	Low	4832 (24)	2574 (24)	133 (3)	25 (9·97)	1·48 (1·10 to 1·99)	1·53 (0·75 to 3·14)	0·009	0·243
	High	4921 (25)	2626 (25)	185 (4)	36 (1)	1·71 (1·29 to 2·28)	1·79 (0·90 to 3·56)	<0·001	0·098
	Highest	4678 (24)	2509 (24)	305 (7)	55 (2)	1·94 (1·47 to 2·56)	1·51 (0·76 to 3·00)	<0·001	0·234
	Missing	690 (4)	380 (4)	16 (2)	3 (0·79)	0·94 (0·54 to 1·63)	1·19 (0·33 to 4·29)	0·832	0·789
**Year of Birth**	-	-	-	-	-	1·07 (1·02 to 1·12)	1·00 (0·90 to 1·11)	0·003	0·949

^1^ counted as “0” if occurred after the conviction

^2^ Highest index of disadvantage represents the lowest level of socioeconomic status (SES)

## Discussion

The results of this retrospective cohort data linkage study are consistent with a modest increase in the risk of offending, including violent offending, following a hospital- documented TBI. The association was found in models adjusted for several important confounders and among siblings discordant for TBI and would be consistent with a causal role for TBI in the subsequent committal of offences.

Previous work suggests a range of possible mechanisms for the well-established association between TBI and offending. First, a TBI might, via sequelae such as behavioural dysregulation, aggression, or impulsivity, be causally implicated in offending behaviour [2, 7]. Our own previous studies suggest that the majority of TBIs reported by convicted offenders are mild, albeit often experienced on multiple occasions. While mild TBI rarely leads to significant cognitive or behavioural changes [19–20], mild TBI *warranting admission to hospital* has been associated with the subsequent development of adverse behaviours[8]. Results from the current study are consistent with such a mechanism.

Second, a TBI might be an independent consequence of a behavioural phenotype that also leads to offending [21]. Certain behaviours in early life that are associated with an increased risk of criminal behaviour in adulthood [22–23] also elevate the risk of head injury [24–25]. In the Dunedin Multidisciplinary Health and Development Study, behavioural ratings were made on children at the age of three. Compared with three year olds who were ‘well-adjusted’, those who were ‘under-controlled’ were, at the age of 21 y, more likely to be diagnosed with antisocial personality disorder, be recidivistic offenders, or have been convicted for a violent offence [22]. In a Finnish study, aggression in the form of bullying at age 8 independently predicted later violent, property and traffic offences [23]. Aggression in early life has also been associated with increased rates of subsequent head trauma and other injuries in childhood and adolescence [24–25]. Heritability of impulsivity, violence, and criminality has been well established in the literature [26–27]. Environmental exposures and genetic similarity is greatest in same sex twins—an ideal comparison group to take account of these potential confounds. In the current study, when siblings served as controls, the estimates of risk of offending associated with TBI were similar to those obtained using population controls, except for sub-analyses in women with small sample sizes or sparse outcomes (twins, violent offending). Within the recognised limitations of family studies (see below), results of the current study provide little support for mechanism two as a major explanation for the association of TBI and offending. Third, a TBI might lead to (or follow from) mental illness [28–29] which for complex and poorly-understood reasons is strongly associated with offending [30]. We repeated the analyses, recoding to include only instances of mental illness recorded prior to the occurrence of TBI (or pseudo TBI). The risk estimates of offending associated with TBI in these supplementary analyses were unchanged, suggesting that mental illness does not mediate risk associated with TBI in a causal pathway to offending. Fourth, a TBI could occur as a consequence of an alcohol/substance use disorder that is the proximate cause for offending [2, 5, 10]. Fifth, criminal activity or incarceration could lead to a TBI, an instance of reverse causation. Prisons are comparatively violent places. This seems unlikely to account for any but a small proportion of the excess TBI prevalence in offender populations, based on our own studies [20, 31]. Finally, other combinations or interactions between mechanisms might occur, e.g. following a TBI, alcohol abuse may have greater behavioural consequences, perhaps leading to offending behaviour.

The results from our analyses using population controls are similar to those of Timonen et al., whose unadjusted risk-estimate for all convictions associated with a TBI was 1·7 [6]. The lack of sibling controls renders their estimate of risk less compelling, in terms of a causal association between TBI and criminality. In relation to violent convictions, the risk estimates related to TBI that we obtained were more modest than those reported by Fazel et al., [7] but there were some important methodological differences that might account for the discrepancies. In the Fazel study, individuals were not excluded from the analyses if they had been convicted of a crime before the occurrence of TBI, although only convictions after the TBI were ‘counted’. This is important because previous criminality may be more prevalent among individuals who sustain a TBI and past criminality is a risk factor for subsequent crime [32]. Interestingly, in a study of US Veterans post deployment, those screen positive for a TBI were no more likely to have a history of subsequent arrest than those screen negative, after adjustment for relevant confounders including previous criminal arrests [33].

In the present study, we excluded from the analyses all individuals with criminal convictions prior to their TBI (with parallel exclusions in the controls), an appropriate strategy, we believe, for evaluating the hypothesis that TBI ‘*causes’* criminal behaviour. Also by contrast with the Fazel study, we adjusted for mental illness as a potential confound and conducted additional analyses using same sex *twin* controls. The study by McKinlay et al. included offending data derived from self-report, psychiatric history was not included as a covariate in the analyses, nor were siblings available as controls although other family factors were considered [8].

Our study had some limitations. Conviction records provide an imprecise measure of *offending behaviour*, given the evidence to suggest that much offending goes undetected and in this respect the McKinlay study has advantages relative to our own [34, 8]. Hospital admission records provide incomplete ascertainment of TBIs, and TBIs in the comparison group, albeit mild, could potentially bias the results toward the null. Despite certain strengths—particularly relating to control for genetic factors—sibling/twin studies have the potential for bias introduced by measurement error or unmeasured confounding, especially for analyses that depend on discordant pair analyses such as in our study [35–36]. For example, substance abusers, at increased risk both for offending and TBI, may be less inclined to seek hospital treatment for mild TBI than non-abusers (i.e. selective under-ascertainment of the exposure), a phenomenon that could bias toward the null and may have contributed to the somewhat unexpected findings in several of the sub analyses in women [37]. Indeed, another study limitation is that our proxy for drug and alcohol abuse did not capture levels of exposure that would be expected and possibly reflect the lack of treatment opportunities in this population. Finally, our results relate to time to first criminal conviction, and TBI as a single binary exposure without stratification by severity, although all those with TBI had been injured sufficiently to warrant hospitalization. Strengths of the study include the cohort size, stratification by sex, inclusion of multiple control groups, including twins of the probands, and our examination of the impact of comorbidity, especially mental illness, on the risk of a conviction.

In conclusion, the results from the current study would be consistent with a causal relationship between TBI and subsequent criminal convictions, and convictions for violence in particular, in both sexes. Successful reduction in the prevalence of TBI, a major public health imperative, could also have benefits in terms of crime rate reduction. Future research directions will include examining the relative contribution of specific factors implicated in the association between TBI and offending, and the development of predictive models that may help to better explain this complex relationship. Gender and ethnic differences, maternal and birth characteristics, and age at TBI may be important in this regard.

## Supporting Information

S1 Table International Classification of Diseases (ICD) 9 and ICD 10 codes used to define Traumatic Brain Injury (TBI). (DOCX)

S2 Table Demographic Characteristics of TBI-Exposed and Community Comparison Males and Females. (DOCX)

S3 Table Demographic Characteristics of TBI-Exposed and Sibling Comparison Males and Females. (DOCX)

S4 Table Demographic Characteristics of TBI-Exposed and Twin Comparison Males and Females. (DOCX)
