# Supplementary material for: Does Traumatic Brain Injury Lead to Criminality? A Whole-Population Retrospective Cohort Study Using Linked Data
Source: PLoS One. 2015 Jul 14;10(7):e0132558. doi: 10.1371/journal.pone.0132558 (PMC4501545; doi:10.1371/journal.pone.0132558)
Supplement: S1 Table — (DOCX) [file pone.0132558.s001.docx]

**Table S1. International Classification of Diseases (ICD) 9 and ICD 10 Codes used to Define Traumatic Brain Injury (TBI)**

| **General Description** | **TBI Codes for Primary Analyses** |
| --- | --- |
| Sequelae (present 1 year or more after the acute injury) of fracture of skull and face bones | T90.2 |
| Sequelae of intracranial injury | T90.5 |
| Fracture of vault of skull | 800, S02.0 |
| Fracture of base of skull | 801, S02.1 |
| Other and unqualified skull fractures | 803, S02.8, S02.9 |
| Multiple fractures involving skull or face with other bones | 804, S02.7 |
| Concussion with varying lengths of loss of consciousness and recovery | 850, 850.1-850.5, 850.9  S06.00-S06.05, S09.09 |
| Cerebral laceration and contusion | 851, S06.38 |
| Subarachnoid, subdural and extradural haemorrhage, following injury | 852, S06.4-S06.6 |
| Other and unspecified intracranial haemorrhage following injury | 853 |
| Diffuse brain injury | S06.2 |
| Focal brain injury | S06.3 |
| Traumatic cerebral oedema | S06.1 |
| Intracranial injury of other and unspecified nature | 854, S06.8, S06.9 |
