# Supplementary material for: Does Traumatic Brain Injury Lead to Criminality? A Whole-Population Retrospective Cohort Study Using Linked Data
Source: PLoS One. 2015 Jul 14;10(7):e0132558. doi: 10.1371/journal.pone.0132558 (PMC4501545; doi:10.1371/journal.pone.0132558)
Supplement: S2 Table — (DOCX) [file pone.0132558.s002.docx]

**Table S2. Demographic Characteristics of TBI-Exposed and Community Comparison Males and Females**

| **Characteristics** | | **Males** | | | **Females** | | |
| --- | --- | --- | --- | --- | --- | --- | --- |
|  |  | **TBI-exposed**  **N=5018, [n (%)]** | **TBI non-exposed**  **N=14904, [n (%)]** | **p value** | **TBI-exposed**  **N=2676, [n (%)]** | **TBI non-exposed**  **N=8007, [n (%)]** | **p value** |
| **Corrective Services record** | **-** | 920 (18) | 1554 (10) | <0.0001 | 238 (9) | 332 (4) | <0.0001 |
| **Drug and Alcohol treatment** | **-** | 24 (0.36) | 54 (0.48) | 0.255 | 17 (0.64) | 32 (0.40) | 0.119 |
| **Mental health problems** | **-** | 929 (19) | 1382 (9) | <0.001 | 596 (22) | 1025 (13) | <0.0001 |
| **Aboriginal status** | **-** | 544 (11) | 870 (6) | <0.0001 | 462 (17) | 510 (6) | <0.0001 |
| **Year of birth** | **1980** | 851 (17) | 2527 (17) | NA^a^ | 477 (18) | 1422 (18) | NA^a^ |
|  | **1981** | 909 (18) | 2693 (18) | NA^a^ | 501 (19) | 1502 (19) | NA^a^ |
|  | **1982** | 850 (17) | 2534 (17) | NA^a^ | 453 (17) | 1354 (17) | NA^a^ |
|  | **1983** | 819 (16) | 2427 (16) | NA^a^ | 423 (16) | 1267 (16) | NA^a^ |
|  | **1984** | 824 (16) | 2455 (16) | NA^a^ | 402 (15) | 1203 (15) | NA^a^ |
|  | **1985** | 765 (15) | 2268 (15) | NA^a^ | 420 (16) | 1253 (16) | NA^a^ |
| **Index of Disadvantage** | **Lowest** | 973 (19) | 3828 (26) | <0.0001 | 483 (18) | 2105 (26) | <0.0001 |
|  | **Low** | 1289 (26) | 3543 (24) | <0.0001 | 619 (23) | 1955 (24) | <0.0001 |
|  | **High** | 1364 (27) | 3557 (24) | <0.0001 | 724 (27) | 1902 (24) | <0.0001 |
|  | **Highest^b^** | 1211 (24) | 3467 (23) | <0.0001 | 748 (28) | 1761 (22) | <0.0001 |
|  | **Missing** | 181 (4) | 509 (3) | <0.0001 | 102 (4) | 278 (4) | <0.0001 |

^a^ Matched by year of birth.

^b^ Highest index of disadvantage represents the lowest level of socioeconomic status (SES)
