# Supplementary material for: Does Traumatic Brain Injury Lead to Criminality? A Whole-Population Retrospective Cohort Study Using Linked Data
Source: PLoS One. 2015 Jul 14;10(7):e0132558. doi: 10.1371/journal.pone.0132558 (PMC4501545; doi:10.1371/journal.pone.0132558)
Supplement: S3 Table — (DOCX) [file pone.0132558.s003.docx]

**Table S3. Demographic Characteristics of TBI-Exposed and Sibling Comparison Males and Females**

| **Characteristics** | | **Males** | | | **Females** | | |
| --- | --- | --- | --- | --- | --- | --- | --- |
|  |  | **TBI-exposed**  **N=1570**  **n (%)** | **TBI non-exposed**  **N=1570**  **n (%)** | **P value** | **TBI-exposed**  **N=827**  **n (%)** | **TBI non-exposed**  **N=827**  **n (%)** | **P value** |
| **Corrective Services record** | | 224 (14) | 152 (10) | <0.0001 | 46 (6) | 38 (5) | 0.37 |
| **Drug and Alcohol treatment** | | 12 (0.8) | 5 (0.3) | 0.08 | 6 (0.7) | 6 (0.7) | 1 |
| **Mental health problems** | | 317 (20) | 176 (11) | <0.0001 | 159 (19) | 104 (13) | <0.0001 |
| **Aboriginal status** | | 128 (8) | 128 (8) | NA | 104 (13) | 104 (13) | NA |
| **Year of birth** | 1980 | 188 (12) | 187 (12) | <0.0001 | 116 (14) | 82 (10) | <0.0001 |
|  | 1981 | 213 (14) | 205 (13) | <0.0001 | 114 (14) | 107 (13) | <0.0001 |
|  | 1982 | 270 (17) | 233 (15) | <0.0001 | 135 (16) | 133 (16) | <0.0001 |
|  | 1983 | 290 (19) | 248 (16) | <0.0001 | 155 (19) | 107 (13) | <0.0001 |
|  | 1984 | 109 (20) | 190 (12) | <0.0001 | 136 (16) | 120 (15) | <0.0001 |
|  | 1985 | 300 (19) | 154 (10) | <0.0001 | 171 (21) | 96 (12) | <0.0001 |
|  | 1986 | - | 153 (10) | <0.0001 | - | 74 (9) | <0.0001 |
|  | 1987 | - | 119 (8) | <0.0001 | - | 69 (8) | <0.0001 |
|  | 1988 | - | 60 (4) | <0.0001 | - | 29 (4) | <0.0001 |
|  | 1989 | - | 15 (1) | <0.0001 | - | 7 (0.8) | <0.0001 |
|  | 1990 | - | 5 (0.3) | <0.0001 | - | 3 (0.4) | <0.0001 |
| **Index of Disadvantage** | Lowest | 426 (27) | 422 (27) | 0.87 | 209 (25) | 204 (25) | 0.95 |
|  | Low | 418 (27) | 418 (27) | - | 229 (28) | 237 (28) | - |
|  | High | 357 (23) | 358 (23) | - | 192 (23) | 187 (23) | - |
|  | Highest^a^ | 307 (20) | 298 (19) | - | 164 (20) | 166 (20) | - |
|  | Missing | 62 (4) | 74 (5) | - | 33 (4) | 39 (5) | - |

^a^ Highest index of disadvantage represents the lowest level of socioeconomic status (SES)
