# Supplementary material for: Does Traumatic Brain Injury Lead to Criminality? A Whole-Population Retrospective Cohort Study Using Linked Data
Source: PLoS One. 2015 Jul 14;10(7):e0132558. doi: 10.1371/journal.pone.0132558 (PMC4501545; doi:10.1371/journal.pone.0132558)
Supplement: S4 Table — (DOCX) [file pone.0132558.s004.docx]

**Table S4. Demographic Characteristics of TBI-Exposed and Twin Comparison Males and Females**

| **Characteristics** | | **Males** | | | **Females** | | |
| --- | --- | --- | --- | --- | --- | --- | --- |
|  |  | **TBI-exposed**  **N=60, n (%)** | **TBI non-exposed**  **N=60, n (%)** | **P value**^a^ | **TBI-exposed**  **N=39, n (%)** | **TBI non-exposed**  **N=39, n (%)** | **P value**^a^ |
| **Corrective Services record** | | 8 (13) | 9 (15) | 1.0 | 1 (3) | 3 (8) | 0.61 |
| **Drug and Alcohol treatment** | | 0 (0) | 0 (0) | - | 0 (0) | 2 (3) | 1.0 |
| **Mental health problems** | | 8(13) | 7 (12) | 0.78 | 9 (23) | 6 (15) | 0.38 |
| **Aboriginal status** | | 2 (3) | 2 (3) | NA**^b^** | 6 (15) | 6 (15) | NA**^b^** |
| **Year of birth** | **1980** | 11 (18) | 11 (18) | NA**^b^** | 4 (10) | 4 (10) | NA**^b^** |
|  | **1981** | 14 (23) | 14 (23) | NA**^b^** | 9 (23) | 9 (23) | NA**^b^** |
|  | **1982** | 8 (13) | 8 (13) | NA**^b^** | 7 (18) | 7 (18) | NA**^b^** |
|  | **1983** | 9 (15) | 9 (15) | NA**^b^** | 4 (10) | 4 (10) | NA**^b^** |
|  | **1984** | 9 (15) | 9 (15) | NA**^b^** | 7 (18) | 7 (18) | NA**^b^** |
|  | **1985** | 9 (15) | 9 (15) | NA**^b^** | 8 (21) | 8 (21) | NA**^b^** |
| **Index of Disadvantage** | **Lowest** | 19 (32) | 19 (32) | NA**^b^** | 7 (18) | 7 (18) | NA**^b^** |
|  | **Low** | 13 (22) | 13 (22) | NA**^b^** | 9 (23) | 9 (23) | NA**^b^** |
|  | **High** | 12 (20) | 12 (20) | NA**^b^** | 11 (28) | 11 (28) | NA**^b^** |
|  | **Highest^c^** | 13 (22) | 13 (22) | NA**^b^** | 11 (28) | 11 (28) | NA**^b^** |
|  | **Missing** | 3 (5) | 3 (5) | NA**^b^** | 1 (3) | 1 (3) | NA**^b^** |

^a^ Fisher’s Exact Test

^b^ will be identical for twins

^c^ Highest index of disadvantage represents the lowest level of socioeconomic status (SES)
